# Brain-targeted heptapeptide-loaded exosomes attenuated ischemia–reperfusion injury by promoting the transfer of healthy mitochondria from astrocytes to neurons

**DOI:** 10.1186/s12951-022-01425-6

**Published:** 2022-05-23

**Authors:** Wei Liu, Chang Su, Yao Qi, Jia Liang, Liang Zhao, Yijie Shi

**Affiliations:** 1 School of Pharmacy, Jinzhou Medical University, Jinzhou, 121000 People’s Republic of China; 2 School of Veterinary Medicine, Jinzhou Medical University, Jinzhou, 121000 People’s Republic of China; 3 Life Science Institution, Jinzhou Medical University, Jinzhou, 121000 People’s Republic of China

**Keywords:** Mitochondria, Ischemic injury, Exosomes, Heptapeptide, Astrocytes

## Abstract

**Background:**

The exchange of mitochondria reportedly plays an important role in cell–cell communication in the central nervous system (CNS). The transfer of fragmented and dysfunctional astrocytic mitochondria into neurons and subsequent mitochondrial fusion often cause serious neuronal damage and cerebral ischaemic injury.

**Methods:**

In this study, we prepared macrophage-derived exosomes laden with heptapeptide (Hep) as a dynamin-related protein-1 (Drp1)–fission 1 (Fis1) peptide inhibitor P110 to alleviate cerebral ischemia–reperfusion injury by reducing mitochondrial Drp1/Fis1 interaction-mediated astrocytic mitochondrial disorder and promoting the transfer of astrocyte-derived healthy mitochondria into neurons.

**Results:**

The results demonstrated that Hep-loaded macrophage-derived exosomes (EXO-Hep) reduced mitochondrial damage in astrocytes by inhibiting the Drp1/Fis1 interaction after ischemia–reperfusion, ensuring the release of heathy astrocytic mitochondria and their subsequent transmission to neurons, alleviating mitochondria-mediated neuronal damage.

**Conclusion:**

EXO-Hep significantly mitigated ischemic injury in a model of transient middle cerebral artery occlusion (tMCAO) by reducing the infarct area and improving neurological performance during the process of cerebral ischemia–reperfusion.

**Graphical Abstract:**

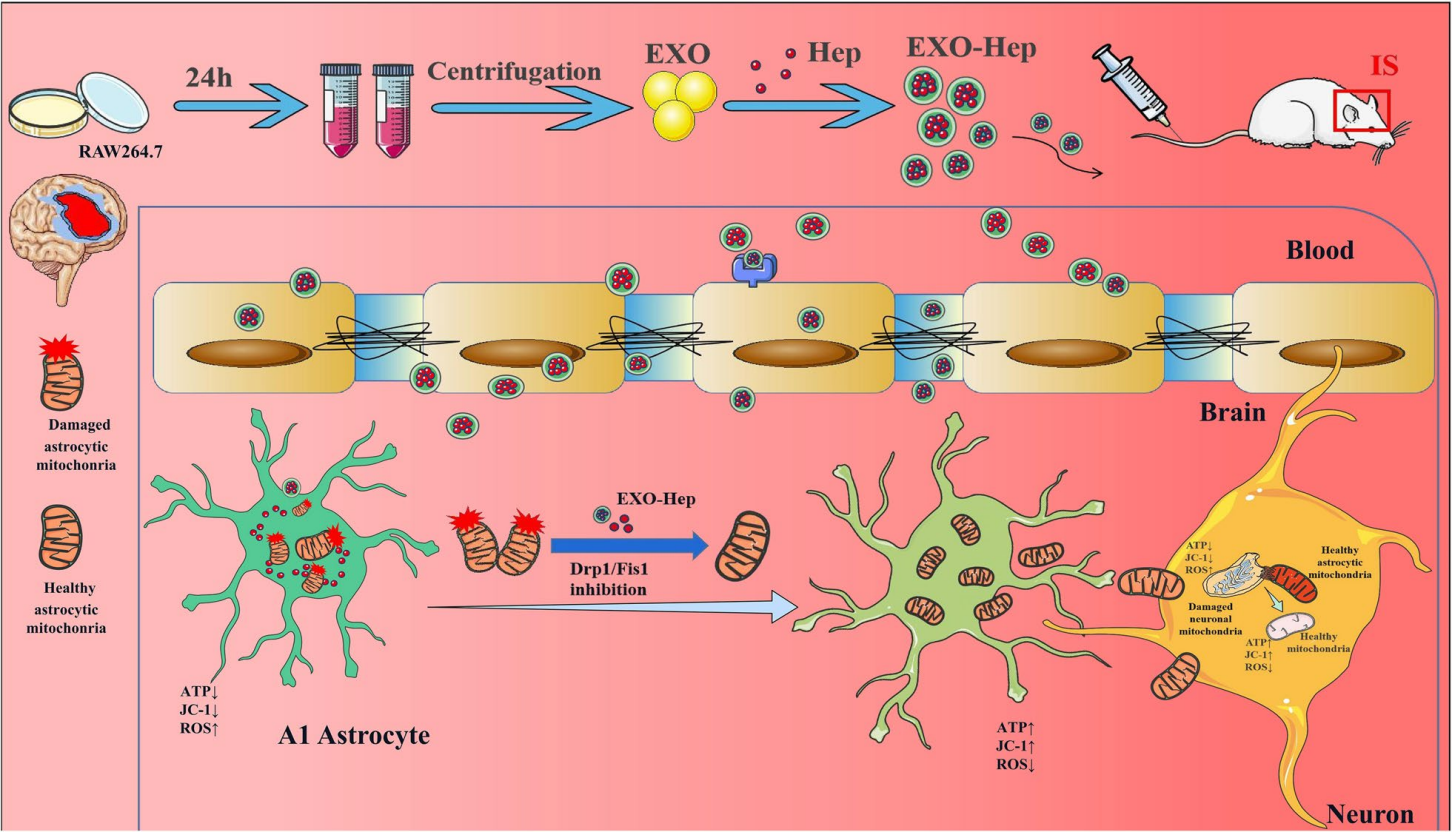

## Background

Ischemic stroke (IS), characterized by a common acute cerebrovascular disorder, is one of the primary fatal diseases in middle-aged and elderly people [1]. However, recent available effective treatment options are limited and only suitable for a small number of stroke patients. Urokinase and streptokinase are neuroprotective drug candidates that have been clinically applied for the treatment of IS. Although these drugs can activate plasminogen to form plasmin, they can also degrade coagulation factors and fibrinogen. Therefore, they potentiate a high risk of bleeding when used in thrombolytic therapy [2]. In addition, United States Food and Drug Administration (FDA) has proved that recombinant tissue plasminogen activator can be used to recanalize blocked vessels in the treatment of IS, however, its therapeutic efficacy is very limited because of a narrow therapeutic window (< 4.5 h) and an increased risk of intracranial hemorrhage [3]. Therefore, it is urgent to develop effective treatments to reduce IS-induced brain injury.

Astrocytes (AS), the supporting matrix cells in the central nervous system (CNS), have multiple regulatory functions, including buffering extracellular ions, clearing amino acid neurotransmitters, limiting excitatory toxicity and promoting synaptic development [4, 5]. Recently, the crosstalk between astrocytes and neurons has been more fully investigated and clarified [6]. On the one hand, IS induces the transition of astrocytes from the resting state to the reactive state and releases cytokines, interleukins and other potentially cytotoxic molecules at higher levels than that from resting astrocytes, thus destroying synaptic homeostasis and initiating neuronal injury [7]. On the other hand, mitochondria can be secreted from astrocytes and delivered into neurons, modulating the function of damaged neuronal mitochondria to affect neuronal damage [8, 9]. Briefly, when IS occurs, astrocytes are rapidly induced to become activated A1-type astrocytes (A1-AS). As a result, under pathological stress, mitochondrial dynamin-related protein-1 (Drp1) as a key mitochondrial regulatory protein predominantly interacts with fission 1 (Fis1), causing an exaggerated fission process, as evidenced by excessive mitochondrial fragmentation, production of reactive oxygen species (ROS) and loss of mitochondrial membrane potential [10]. Therefore, marked pathological fragmentation of mitochondria is produced, and mitochondrial function is significantly disrupted by decreasing adenosine triphosphate (ATP) and mitochondria membrane potential [11]. Finally, damaged astrocytic mitochondria are further released, enter adjacent neurons and induce fusion with neuronal mitochondria, inducing neuronal mitochondrial dysfunction, amplifying neuronal damage and worsening neurological outcomes [12, 13]. In contrast, after incubation of normal astrocytic mitochondria with oxygen–glucose deprivation (OGD)-injured neurons, astrocytic mitochondria can be detected in neurons, restore ATP levels in injured neurons and enhance cell survival and plasticity [14]. These findings indicate a new mitochondrial crosstalk between astrocytes and neurons that may contribute to neurological regulation after IS. Improving the function of astrocytic mitochondria and promoting the delivery of healthy astrocytic mitochondria into neurons are potential therapeutic targets for reducing IS-induced neuronal injury and neurodegenerative disorders.

**Fig. 1:**
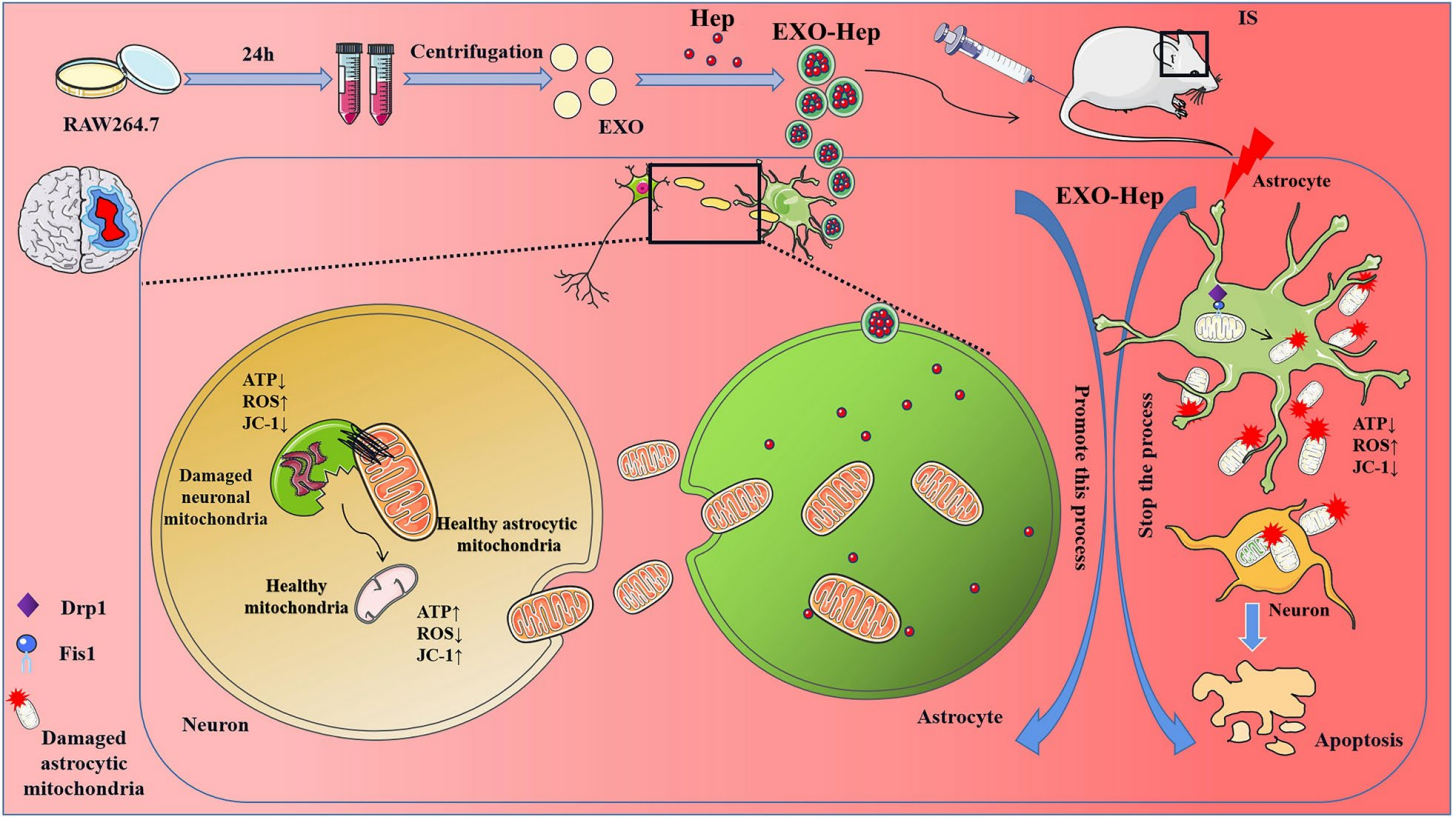
The primary hypothesis of this study. We fabricated brain-targeted multifunctional biomimetic heptapeptide loaded exosomes for the treatment of IS. Heptapeptide loaded macrophage derived exosomes (EXO-Hep) targeted brain and inhibited Drp1/Fis1 interaction to improve the mitochondrial function of astrocyts. As a result, more healthy astrocytic mitochondria were secreted from astrocytes and transferred into neurons for reducing mitochondria-mediated damage of neurons. Finally, EXO-Hep ameliorated IS injury by reducing infarct area and improving neurological performance in tMCAO rats

To treat IS by modulating mitochondrial crosstalk between astrocytes and neurons, we focused on inhibiting Drp1/Fis1-mediated mitochondrial fission in type A1 astrocytes to reduce the transfer of damaged astrocytic mitochondria into neurons. Heptapeptide (Hep), a Drp1–Fis1 peptide inhibitor P110, alleviates mitochondrial dysfunction and is a key contributor in IS [15]. It selectively inhibits the binding of activated Drp1 to Fis1 and reduces the pathological fission of astrocytic mitochondria without affecting physiological fission [16]. Therefore, treatment with Hep has been shown to be beneficial for reducing IS mediated damage in vitro and in vivo [17]. However, Hep as a protein drug suffers the susceptibility to enzymatic degradation, short circulation half-lives and poor membrane permeability, thus posing significant barriers for effective delivery [18]. To overcome these shortcomings to convey a better therapeutic effect, various carriers, such as liposomes, polymer vesicles, exosomes, dendrimers, and inorganic nanoparticles, have been applied to encapsulate drugs to establish a protein drug-targeted delivery system [19]. Among these carriers, exosomes characterized by low immunogenicity, high biological permeability, and high delivery efficiency have promising potential innate properties for loading protein agents and targeting ischemic regions [20]. As shown in Fig. 1, we prepared Hep-loaded macrophage-derived exosomes (EXO-Hep) to attenuate mitochondrial disorder in astrocytes by inhibiting the Drp1/Fis1 mediated mitochondrial fission in astrocytes. Subsequently, more healthy mitochondria of astrocytes were released and transferred into neurons, resulting in reduced neuronal damage by improving neuronal mitochondrial function. Finally, EXO-Hep alleviated cerebral ischemia–reperfusion injury in a model of transient middle cerebral artery occlusion (tMCAO).

### Materials and animals

Hep, as the Drp1 inhibitor P110 was synthesized from Nanjing Peptide Industry Biotechnology Co., Ltd, PKH26 was obtained from Sigma-Aldrich Co. Antibodies such as Drp1, TOM20, BAX, BCL-2, Fis1 (1:500), were purchased from WanLeiBio (Shenyang, China), and rabbit anti-β-actin was obtained (1:1000, Biogot Technology, Co, Ltd). This study acquired goat anti-rabbit IgG/HRP secondary antibody (1:10,000) from EarthOx Life Sciences (Millbrae, CA, USA). All reagents and chemicals were provided by Sigma (St. Louis, MO, USA).

The murine macrophage RAW264.7 cell line was purchased from the Type Culture Collection of Chinese Academy of Sciences (Shanghai, People’s Republic of China) and cultured in RPMI 1640 medium (HyClone, UT, USA) containing 10% foetal bovine serum (FBS; Gibco, CA, USA) and 1% penicillin–streptomycin (PS). SH-SY5Y cells were purchased from the Type Culture Collection of Chinese Academy of Sciences (Shanghai, People’s Republic of China) and cultured in Dulbecco’s Modified Eagle’s Medium/Nutrient Mixture F-12 (DMEM/F-12) supplemented with 10% foetal bovine serum (FBS) and 1% PS. The astrocyte cell line HA-1800 was purchased from Guang Zhou Jennio Biotech Co., Ltd. and cultured in Dulbecco’s Modified Eagle’s Medium: Nutrient Mixture F-12 (DMEM/F-12) supplemented with 10% of fetal bovine serum (FBS) and 1% PS. To simulate the astrocytic A1 state that occurs in stroke, astrocytes were treated with lipopolysaccharide (LPS) to obtain type A1 astrocytes (A1-AS). Male Sprague–Dawley (SD) rats (250–280 g) were provided by Jinzhou Medical University. The experimental protocol was performed with the approval of the Institutional Animal Care and Use Committee of Jinzhou Medical University and followed the National Guidelines for Animal Protection.

### Preparation and identification of EXO-Hep

According to the previous report [21], RAW 264.7 cells cultured in full growth medium were seeded into culture plates (1 × 10^6^ cells). Twenty-four hours later, the culture medium was collected and filtered. After centrifugation at 20,000×*g* for 30 min, the supernatant was discarded, and exosome pellets were resuspended in phosphate-buffered saline (PBS) and ultracentrifuged again at 100,000×*g* for 150 min to obtain EXO. To load Hep into EXO, 1 mg/mL Hep was added to 8 mg/mL EXO, and the mixture was sonicated using a sonic dismembrator for 4 cycles of 45 s on/2 min off with a 3 min cooling period between each cycle. After sonication, the mixture was incubated in an ice water bath at 4 °C for 24 h. After centrifugation, EXO-Hep was collected and the remaining Hep in the supernatant was measured by checking its UV absorbance. Encapsulation efficacy (EE) of Hep in EXO was calculated as follow:

$$EE\% = \frac{{W_{total} - W_{free} }}{{W_{total} }} \times 100\%$$

$W_{total}$ indicated the amount of initial added Hep, while $W_{free}$ was the amount of Hep remaining in the supernatant.

Identification of EXO was performed by western blotting using anti-Alix (1:2000, Abcam, Cambridge, UK) and anti-CD63 (1:1000, Bioworld, Bloomington, USA) antibodies. The morphology and shape of EXO and EXO-Hep were determined using transmission electron microscopy (TEM, HITACHI HT7800; Hitachi, Ltd). Particle size was measured using a Zetasizer Nano ZS instrument.

### Mitochondria membrane potential measurement

To monitor mitochondrial health, JC-1 dye (Solarbio, Cat no #M8650) was used to assess mitochondrial membrane potential. Mitochondria from astrocytes media and SH-SY5Y cells were incubated with JC1 (5 μM or 1 μM) for 30 min at 37 °C. JC1 dye exhibited potential-dependent accumulation in mitochondria, indicated by fluorescence emission shift from green (Ex 485 nm/Em 516 nm) to red (Ex 579 nm/Em 599 nm). Mitochondria membrane potential was determined by calculating the fluorescent ratio with a fluorescent microplate reader.

### ATP measurement

Relative intracellular or extracellular ATP levels were determined using ATP Luminescent Cell Viability Assay Kit (US EVERBRIGHT, Cat no #A6103S), which can perform cell lysis and generate a luminescent signal proportional to the amount of ATP present [16]. In brief, for intracellular ATP levels, 96-well plates with cell lysate (50 μl) were prepared. An equal volume of the single-one-step reagent provided by the kit was added to each well and incubated for 30 min at room temperature. For measuring ATP content in extracellular mitochondria, cell supernatant was cleared of cellular debris by centrifugation at 1000×*g* for 10 min and then centrifuged 13,000×*g* for 25 min followed by a wash with 1 mL of PBS. The pellet was then resuspended in the 50 μl of serum-free phenol-free DMEM or PBS before an equal volume of the single-one-step reagent provided by the kit with incubation for 30 min at room temperature. ATP content was measured using microplate Reader.

### Determination of ROS production

To determine mitochondrial ROS production [17], extracellular mitochondria were treated with 5 μM MitoSOX™ Red mitochondrial superoxide indicator (Invitrogen, Cat no #40778ES50) at the end of the experiment for 20 min at 37 °C according to the manufacturers’protocols. Fluorescence was analyzed at excitation/emission maxima of 510/588 nm. To determine cellular ROS production, cells were incubated with 5 μM CellROX™ oxidative stress reagent (Invitrogen, Cat no #BB-47053) for 30 min along. The fluorescence was analyzed using microplate reader, at excitation/emission maxima of 485/520 nm for CellROX™.

### MTT and TUNEL assays

Hep, EXO and EXO-Hep were added to the plates and incubated with A1-AS for 24 h to obtain Hep-treated A1-AS (A1-AS (Hep)), EXO-treated A1-AS (A1-AS (EXO)) and EXO-Hep-treated A1-AS (A1-AS (EXO-Hep)). According to a previous report [16], the culture medium (CM) from A1-AS treated with Hep, EXO and EXO-Hep was removed, and the released mitochondria were collected from the culture medium of A1-AS treated with Hep (Mito/A1-AS (Hep)), EXO (Mito/A1-AS (EXO)) and EXO-Hep (Mito/A1-AS (EXO-Hep)). After the astrocytic mitochondria and culture medium were co-incubated with OGD-stimulated SH-SY5Y cells for 24 h, serum-free medium containing MTT was added and incubated for 4 h. Finally, dimethyl sulfoxide was added to OGD-stimulated SH-SY5Y cells, and absorbance was measured at 490 nm. The terminal deoxynucleotidyl transferase-mediated dUTP nick end labelling (TUNEL) assay was also used to detect apoptotic cell level.

### Distribution of EXO-Hep in ischemic brain tissue

To evaluate the location of EXO and EXO-Hep in astrocytes of the ischemic brain, astrocytes were stimulated with LPS followed by the addition of PKH26-labelled EXO and PKH26-labelled EXO-Hep. The distribution of PKH26-labelled EXO and PKH26-labelled EXO-Hep in activated astrocytes was observed by confocal laser scanning microscopy. To evaluate the ability of EXO-Hep to target ischemic brains in vivo, tMCAO rats were intravenously injected with PKH26-labelled EXO-Hep through the tail vein. One hour later, tMCAO rats were first perfused with PBS for removing the blood and the brains of rats were dissected. An IVIS Spectrum imaging system (PerkinElmer, Waltham, MA, USA) was employed to capture PKH26-labelled EXO-Hep-emitted fluorescence images in the brain. Two hours after administration, tMCAO rats were sacrificed and the brains were cryosectioned. After staining with a C3 antibody to label type A1 astrocytes, the colocalization of PKH26-labelled EXO-Hep in A1 astrocytes was investigated using confocal laser scanning microscopy.

### Treatment administration following tMCAO

To establish tMCAO, a 6–0 nylon monofilament suture was inserted into the right internal carotid arteries of SD rats for 2 h and subsequently removed to allow blood reperfusion. After reperfusion, 1 mL PBS, Hep, EXO and EXO-Hep containing Hep at a concentration of 0.75 mg/mL were administered via a single intravenous injection via the tail vein.

### Neurological evaluation of EXO-Hep in the tMCAO model

Twenty-four hours after administration of 1 mL PBS, Hep, EXO and EXO-Hep containing Hep at a concentration of 0.75 mg/mL to SD rats subjected to tMCAO, neuroprotective effects were evaluated by performing TTC (2,3,5-triphenyltetrazolium chloride, Sigma–Aldrich) staining and determining neurological scores and immunofluorescence according to previous reports [22].

### Immunofluorescence staining

Frozen sections of rat brains were kept at room temperature for 30 min. Then, sections were pre-incubated with goat serum followed by primary anti-C3 (1:100, ZenBio Antibody) and anti-NeuN (1:100, Biolegend Antibody) antibodies overnight at 4 °C. After several rinses with PBS, the sections were incubated with secondary antibody (Texas red-conjugated donkey anti-rabbit IgG or fluorescein isothiocyanate [FITC]-conjugated donkey anti-rat IgG) for 1 h. Then, the sections were incubated with DAPI for 5 min. Images were obtained under a fluorescence microscope.

### Western blot assay

Based on a previously reported protocol [23], proteins were extracted from mitochondria, cells and culture medium, and transferred to polyvinylidene fluoride (PVDF) membranes (BioTrace; Pall Corporation, New York, USA). After incubation with primary antibodies such as TOM20, Drp1, Fis1, BAX, BCL-2 (1:500, Wanleibio, Shenyang, China), VDAC (1:1000, Wanleibio, Shenyang, China), β-actin (1:1000, Biogot Technology, Co, Ltd), C3 and cytochrome c (1:1000, Biolegend Antibody), membranes were incubated with a 1:10,000 dilution of the secondary antibody for 1 h at room temperature. The levels of the target proteins were imaged and analysed using a UVP gel analysis system (iBox Scientia 600; UVP, LLC, CA, USA).

### Statistical analysis

The results are presented as the means ± standard deviations (SD). Statistical analyses were performed using one-way analysis of variance (ANOVA). P < 0.05 (*), P < 0.01 (**), P < 0.001 (***) were considered statistically significant differences.

## Results

### Characterization of EXO-Hep

**Fig. 2:**
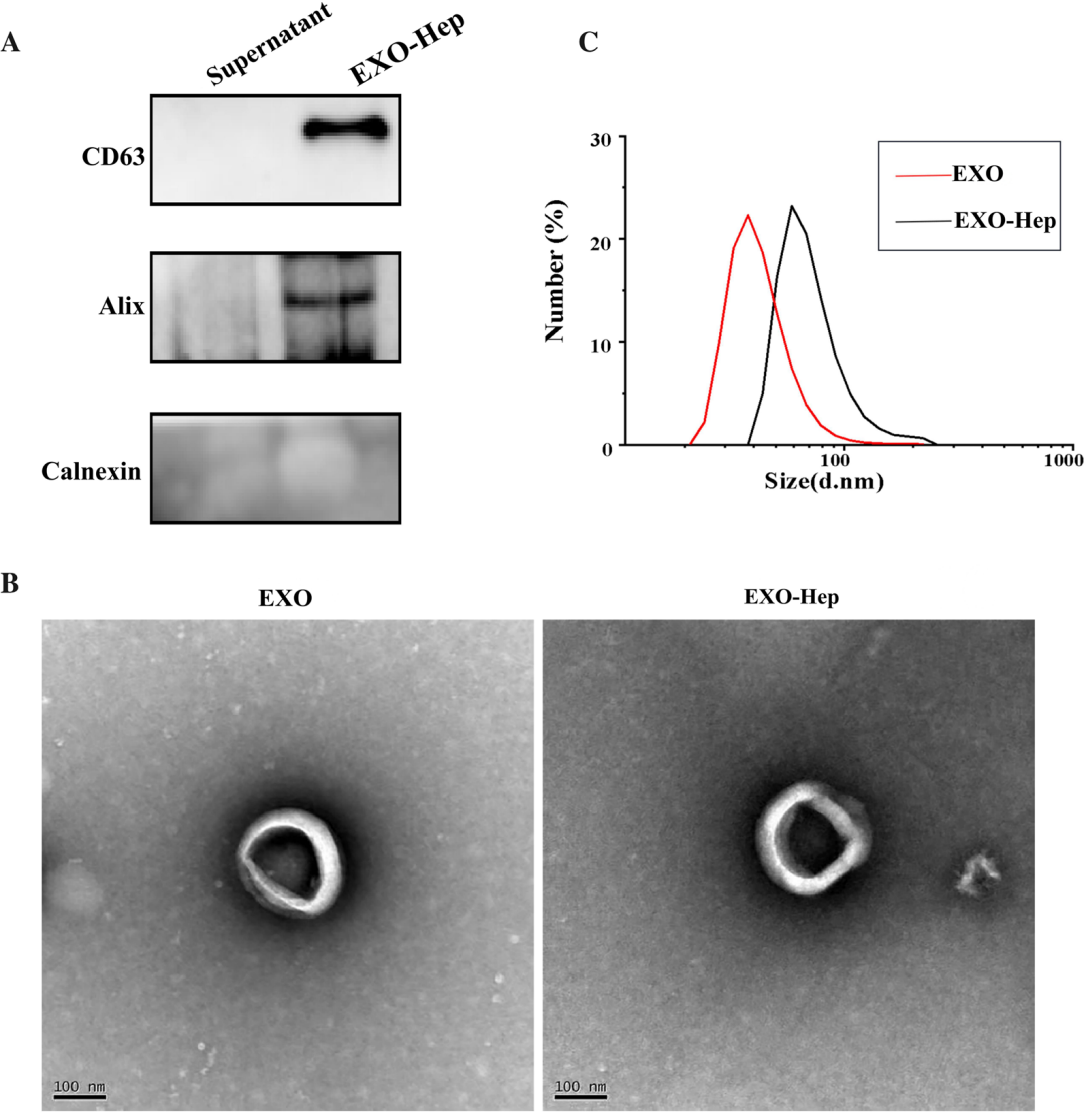
Characterization of EXO and EXO-Hep. **A** Western blotting analysis of exosomes surface markers. EXO was isolated and collected by centrifuging culture medium of RAW264.7 cells. The bottom sediment was collected to obtain EXO and the residual supernatant containing no exosomes was selected as the negative control. Hep was loaded into EXO using sonication and EXO-Hep were obtained by a series of centrifugations. **B** Size and morphology of EXO and EXO-Hep observed by TEM. Scale bar: 100 nm. **C** Particle size distribution of EXO and EXO-Hep measured by DLS

EXO were isolated and collected from culture medium of RAW264.7 cells and the residual supernatant containing no exosomes was selected as the negative control. Hep was loaded into EXO using sonication and EXO-Hep were obtained by a series of centrifugations. As shown in Fig. 2A, exosome-specific markers, such as Alix and CD63, were expressed in EXO-Hep, indicating that EXO-Hep showed the characterization of exosomes with the expressions of exosome-specific markers. When the morphology and particle size of EXO and EXO-Hep were examined using TEM and nanoparticle size tracking analysis, the results revealed that both EXO and EXO-Hep exhibited a typical cup structure (Fig. 2B), and the size of most EXO and EXO-Hep was smaller than 100 nm (Fig. 2C). We also obtained the encapsulation efficiency for Hep of 36.4%.

### EXO enhanced the delivery of Hep in ischemic region in vivo and in vitro

**Fig. 3:**
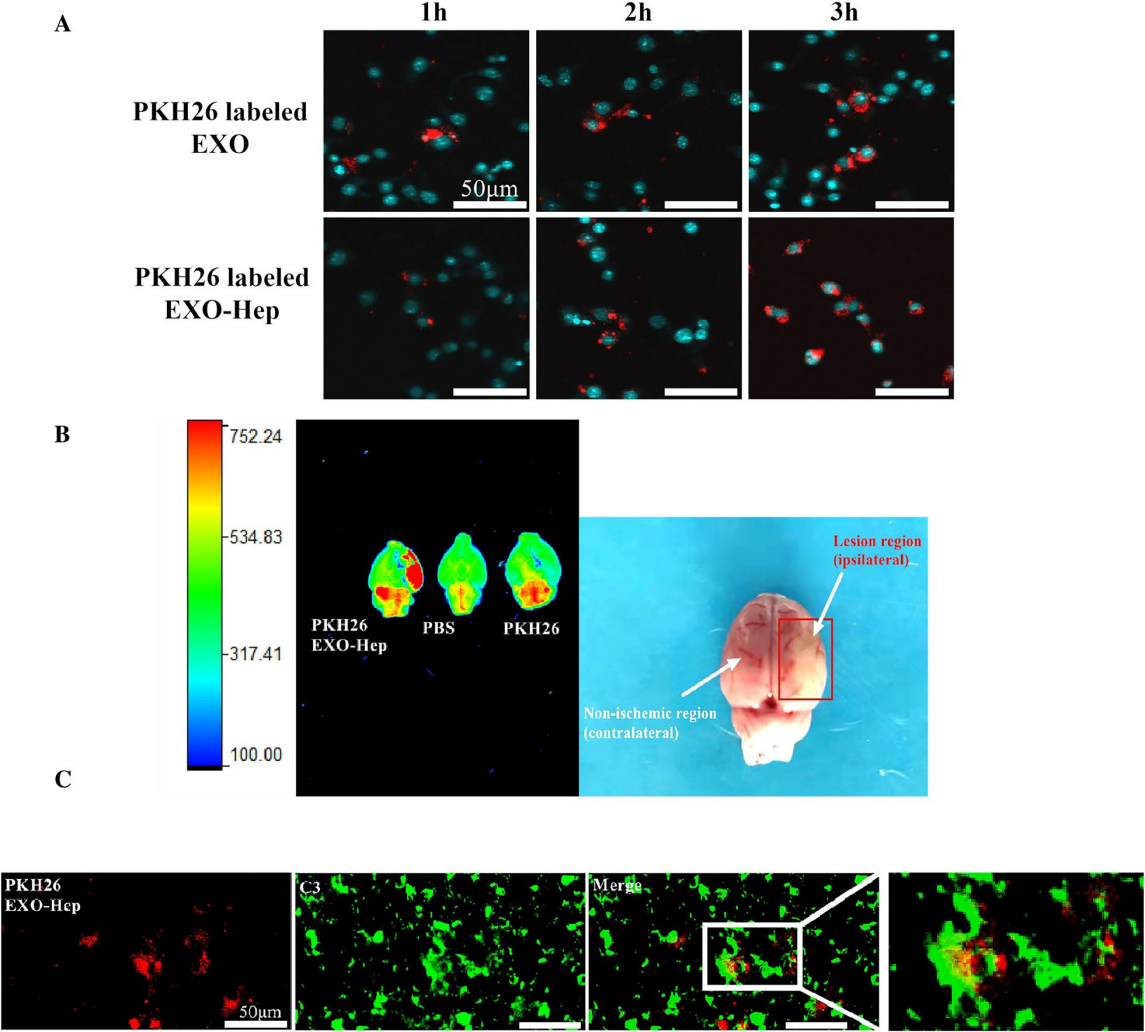
EXO enhanced the accumulation of Hep in the ischemic region in vivo and in vitro*.* **A** Confocal images of A1 astrocytes after incubating with EXO and EXO-Hep. Nucleus was stained with Hoechst 33,342 (blue) for 15 min at 37 °C, and both of EXO and EXO-Hep were labelled by PKH26 (red). The scale bar is 50 μm. **B** Representative fluorescence images of brains of tMCAO rats treated with PBS, free PKH26 and PKH26 labeled EXO-Hep via intravenous injection. **C** Representative fluorescent images of Co-location of PKH26 labelled EXO-Hep (red) with A1 astrocytes (green) at ischemic area of brains from rats receiving 2 h of intravenous administration of PHK26 labelled EXO-Hep. A1 type astrocytes were stained with anti-C3 (green). The scale bar is 50 μm

To evaluate whether EXO-Hep was internalized by A1 astrocytes, LPS was used to stimulate astrocyte activation into stroke-like type A1 astrocytes, followed by the addition of PKH26-labelled EXO-Hep and PKH26-labelled EXO. Confocal laser scanning microscopy revealed that the red fluorescence intensity in A1 astrocytes was gradually enhanced over time (Fig. 3A), suggesting that EXO-Hep was internalized into A1 astrocytes to enhance the accumulation of Hep. We further investigated the targeting ability of EXO-Hep in the ischemic brains of tMCAO rats. After 2 h of cerebral ischemia, suture was removed and PKH26-labelled EXO-Hep was intravenously injected via the tail vein of tMCAO rats. As shown in Fig. 3B, fluorescence imaging results showed enhanced accumulation of PKH26-labelled EXO-Hep, exhibiting much higher fluorescence signals in the ischemic site of the brain than that in the nonischemic hemisphere of the brain, demonstrating that EXO specifically localized Hep at the ischemic site through BBB to increase Hep content in vivo. To further evaluate the specific delivery of EXO-Hep into type A1 astrocytes in the ischemic area, activated type A1 astrocytes were immunostained with a C3 antibody, and the colocalization of PKH26-labelled EXO-Hep in A1 astrocytes was assessed. As shown in Fig. 3C, the red fluorescence emitted by PKH26-labelled EXO-Hep was merged well with the green fluorescence emitted by C3, a marker of A1 astrocytes. This observation indicated that EXO-Hep transported Hep to activated type A1 astrocytes to exert its curative effect. Taken together, these data demonstrated that EXO-Hep help Hep cross the BBB to target the ischemic brain and achieved the distribution of Hep in activated astrocytes.

### EXO-Hep improved neuronal mitochondrial function by inhibiting Drp1/Fis1-mediated mitochondrial fission in type A1 astrocytes and promoting the transfer of healthy astrocytic mitochondria into neurons

**Fig. 4:**
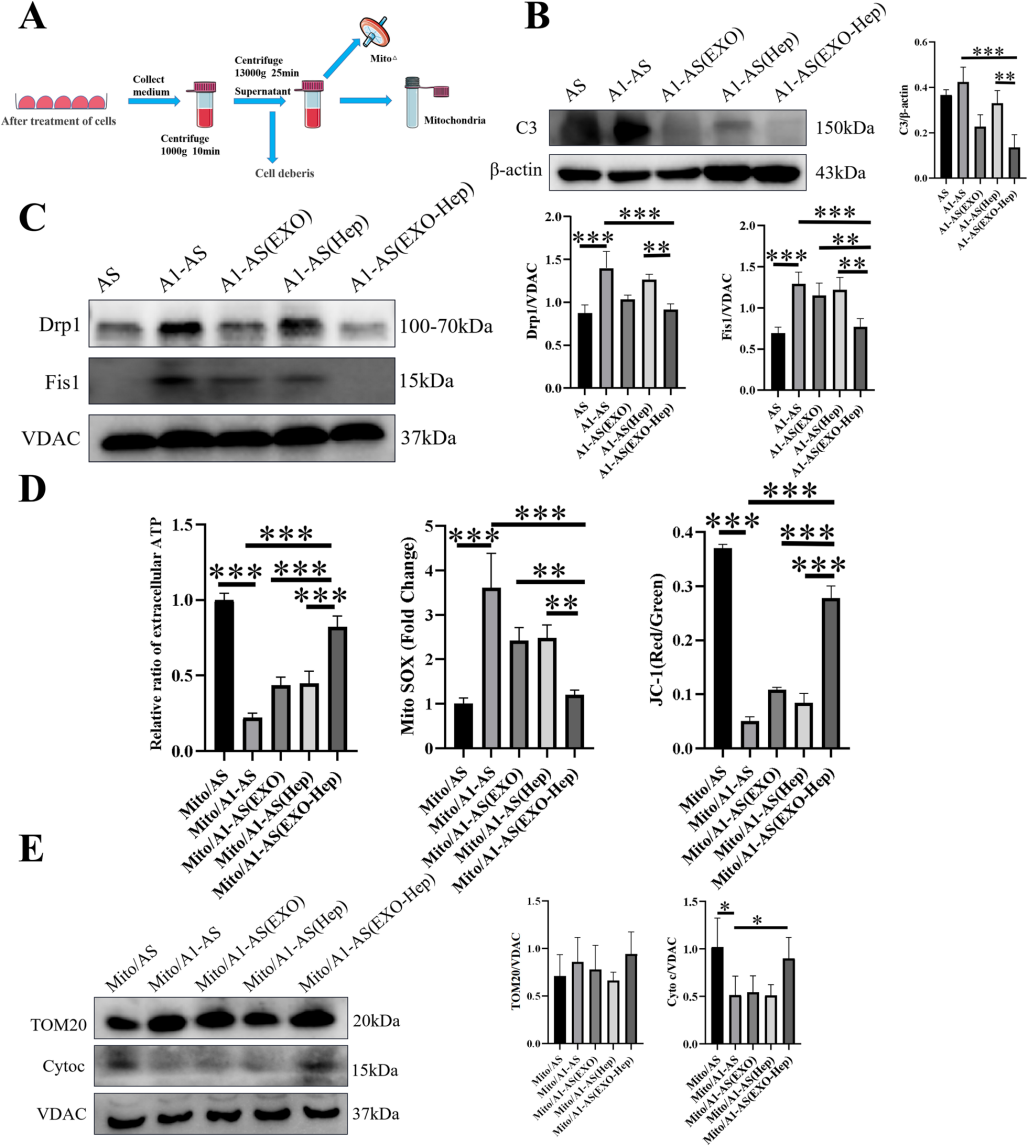
EXO-Hep improved mitochondrial function by inhibiting Drp1 / Fis1 mediated mitochondrial fission in type A1 astrocytes and enhancing the secretion of healthy astrocytic mitochondria. **A** The protocol for obtaining purified mitochondria in the culture medium. **B** A1 astrocytic activation was examined by detecting the expression of C3 (type A1 astrocyte marker) in different groups. Data represent means ± SD (n = 3), **P < 0.01, ***P < 0.001. **C** Western blotting assays on the expressions of Drp1 and Fis1 in intracellular mitochondria of type A1 astrocytes treated with Hep (A1-AS (Hep)), EXO (A1-AS (EXO)) and EXO-Hep (A1-AS (EXO-Hep)). Data represent means ± SD (n = 3), **P < 0.01, ***P < 0.001. **D** Extracellular ATP levels, mitochondrial ROS, and mitochondrial membrane potential, were determined in released Mito/AS, Mito/A1-AS, Mito/A1-AS(Hep), Mito/A1-AS(EXO) and Mito/A1-AS(EXO-Hep). The relative ratio of extracellular ATP was by calculating the ratio of level of ATP in groups to level of ATP in released Mito/AS group. Data represent means ± SD (n = 3), **P < 0.01, ***P < 0.001. **E** Integrity of the released Mito/AS, Mito/A1-AS, Mito/A1-AS (Hep), Mito/A1-AS (EXO) and Mito/A1-AS (EXO-Hep) was determined using western blot for detecting markers of outer-membrane proteins (TOM20) and cytochrome c as an inter-membrane space protein in the released mitochondria, the mitochondrial reference VDAC was used as a reference. Data represent means ± SD (n = 3),*P < 0.05

The role of mitochondria in nonneuronal cell types is recognized to play crucial roles in neuronal regulation [24]. When IS occurs, astrocytes transit from the resting state to the active and neuroinflammatory A1 state, and the interaction between Drp1 and Fis1 is activated to induce pathologic fission and mitochondrial injury. As a result, injured mitochondria was rapidly released from astrocytes and transferred to neurons, thus contributing to detrimental effects on neuronal survival [25]. Therefore, we first explored whether EXO-Hep inhibited Drp1/Fis1-mediated stroke-induced astrocytic activation and improved the secretion of healthy mitochondria. To obtain stroke-like type A1 astrocytes, LPS was added into the cell culture dishes to stimulate type A1 astrocytes according to the previous report [26]. As shown in Fig. 4B and C, compared with normal astrocytes, LPS induced A1 activation in astrocytes by increasing levels of C3, and the Drp1/Fis1 interaction was significantly activated by enhancing the expression of Drp1 and Fis1 in mitochondria of astrocytes for causing exaggerated fission process. Subsequently, dysfunctional mitochondria from A1 astrocytes were released, as evidenced by decreased levels of ATP, loss of mitochondrial membrane potential, massive ROS production and excessive loss of cytochrome c (a marker for mitochondrial damage) in mitochondria (Fig. 4D and E). When Hep, EXO and EXO-Hep were used to treat type A1 astrocytes, levels of C3 were decreased, the Drp1/Fis1 interaction was inhibited by EXO-Hep, resulting in limited mitochondrial fission and reduced formation of damaged mitochondrial fragmentation (Fig. 4C). In addition, mitochondrial ATP levels and membrane potential were significantly enhanced, the generation of extracellular mitochondrial ROS was inhibited and the expression of cytochrome c was enhanced in secreted astrocytic mitochondria (Fig. 4D and E). All results demonstrated that EXO-Hep inhibited the activation of astrocytes and reduced mitochondrial disorder of astrocytes, enabling more healthy mitochondria to be released from astrocytes.

**Fig. 5:**
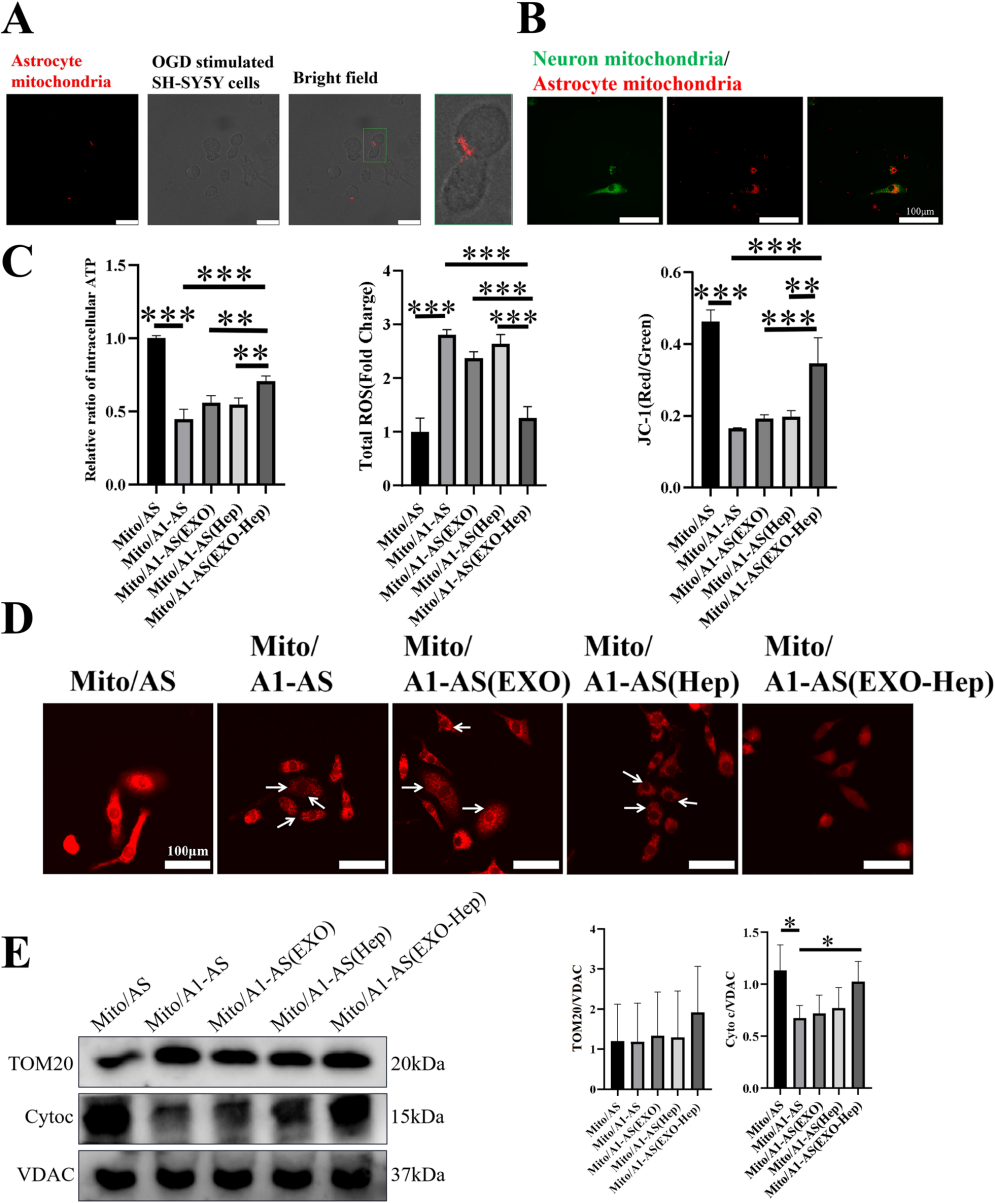
EXO-Hep improved neuronal mitochondrial function by enhancing the transfer of healthy astrocytic mitochondria into neurons. **A** Images of location of astrocytic mitochondria (red) in neurons. The scale bar is 25 μm. **B** Confocal images of fusion between astrocyte mitochondria (red) and neuronal mitochondria (green). The scale bar is 100 μm. **C** ROS, ATP and JC-1 determination in OGD stimulated SH-SY5Y cells treated with Mito/AS, Mito/A1-AS, Mito/A1-AS(Hep), Mito/A1-AS(EXO) and Mito/A1-AS(EXO-Hep). The relative ratio of intracellular ATP in OGD stimulated SH-SY5Y cells was determined by calculating the ratio of level of ATP in OGD stimulated SH-SY5Y cells treated with Mito/AS, Mito/A1-AS, Mito/A1-AS(Hep), Mito/A1-AS(EXO) and Mito/A1-AS(EXO-Hep) to level of ATP in OGD stimulated SH-SY5Y cells treated with Mito/AS. Data represent means ± SD (n = 3), **P < 0.01, ***P < 0.001. **D** Mito Tracker Red staining images of OGD stimulated SH-SY5Y cells treated with Mito/AS, Mito/A1-AS, Mito/A1-AS (Hep), Mito/A1-AS (EXO) and Mito/A1-AS (EXO-Hep). The scale bar is 100 μm. **E** The mitochondrial membrane integrity of OGD stimulated SH-SY5Y cells treated with Mito/AS, Mito/A1-AS, Mito/A1-AS (Hep), Mito/A1-AS (EXO) and Mito/A1-AS (EXO-Hep) was analyzed by determining the expression of TOM20 and cytochrome c in mitochondria of OGD stimulated SH-SY5Y cells, and the mitochondrial reference VDAC was used as a control. Data represent means ± SD (n = 3),*P < 0.05

We next explored our previous hypothesis that mitochondrial dysfunction in astrocytes may regulate the biological function of neurons by transmission of astrocytic mitochondria to neurons and fusion with neuronal mitochondria. After co-incubation of OGD-stimulated SH-SY5Y cells with Mito/AS, Mito/A1-AS, Mito/A1-AS (Hep), Mito/A1-AS (EXO) and Mito/A1-AS (EXO-Hep), colocalization and fusion between released astrocytic mitochondria and neuronal mitochondria were assessed, and the biological function of neuronal mitochondria was investigated by detecting the levels of ATP, mitochondrial membrane potential and production of ROS. Confocal laser scanning microscopy assays (Fig. 5A) demonstrated red fluorescence emitted by MitoTracker Red-labelled astrocytic mitochondria was observed in neurons. Furthermore, we found that red fluorescence emitted by astrocytic mitochondria labelled with MitoTracker Red merged well with the green fluorescence emitted by MitoTracker Green, a marker of neuronal mitochondria (Fig. 5B), indicating obvious colocalization of astrocytic mitochondria with neuronal mitochondria. These results all suggested that mitochondria secreted by astrocytes can enter neurons and further fuse with neuronal mitochondria. Finally, after continuous mitochondrial fusion between astrocytes and neurons, changes in the mitochondrial function of neurons were assessed. As shown in Fig. 5C and E, mitochondria from A1 astrocytes aggravated mitochondrial damage in neurons by decreasing ATP levels, lowering mitochondrial membrane potential, triggering the generation of intracellular ROS and increasing the loss of cytochrome c in mitochondria of neurons. In particular, EXO-Hep-mediated improvement of astrocytic mitochondrial function contributed to the mitochondrial normalization of OGD-stimulated SH-SY5Y cells by increasing ATP levels and mitochondrial membrane potential, inhibiting the generation of intracellular ROS and elevating the level of mitochondrial cytochrome c in mitochondria of neurons. As shown in Fig. 5D, compared with mitochondria of neurons treated with mitochondria isolated from normal astrocytes, excessive mitochondrial fragmentation was induced in mitochondria of neurons treated with released mitochondria from A1 astrocytes, as evidenced by small, round or dot-like staining patterns. In contrast, after co-incubation with mitochondria from A1 astrocytes pre-treated with EXO-Hep, these mitochondrial structure abnormalities in neurons was significantly reduced and restored to mitochondrial interconnectivity and length. Taken together, these findings demonstrated that EXO-Hep mediated the secretion of healthy astrocytic mitochondria and promoted the transfer and fusion of healthy mitochondria between astrocytes and neurons, attenuating mitochondrial deficits in OGD stimulated SH-SY5Y cells.

### EXO-Hep mitigated the OGD induced injury in SH-SY5Y cells by promoting the transfer of healthy astrocytic mitochondria into neurons

**Fig. 6:**
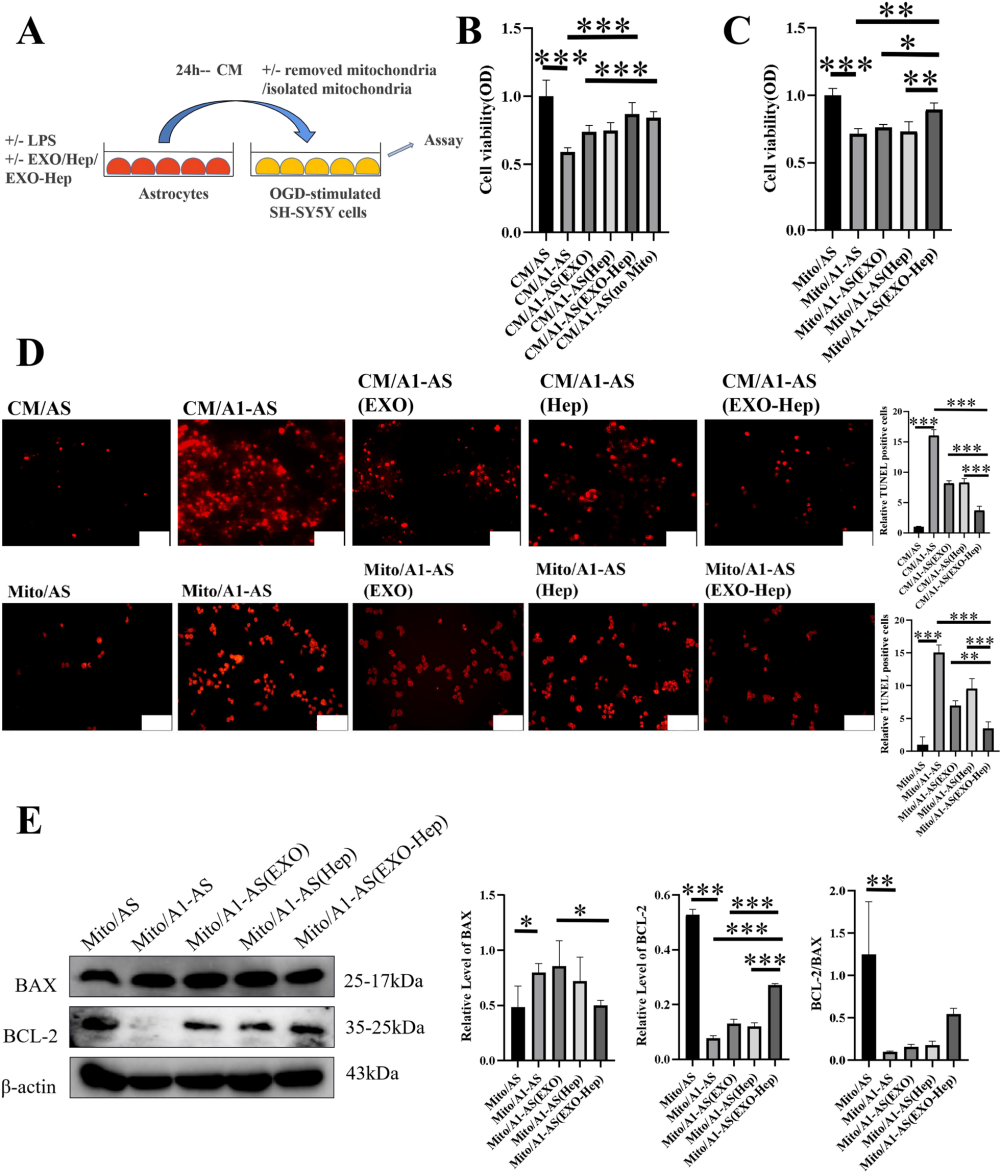
EXO-Hep reduced the mitochondria mediated damage to OGD-stimulated SH-SY5Y cells by promoting transfer of healthy mitochondria of astrocytes into neurons. **A** Schematic diagram of co-incubation of neurons with culture medium of astrocyte and purified astrocytic mitochondria. **B** The cell viability of OGD-stimulated SH-SY5Y cells incubating with CM/AS, CM/A1-AS, CM/A1-AS (EXO), CM/A1-AS (Hep) and CM/A1-AS (EXO-Hep). Data represent means ± SD (n = 3), ***P < 0.001. **C** The cell viability of OGD-stimulated SH-SY5Y cells incubating with Mito/AS, Mito/A1-AS, Mito/A1-AS (EXO), Mito/A1-AS (Hep) and Mito/A1-AS (EXO-Hep). Data represent means ± SD (n = 3),*P < 0.05, **P < 0.01, ***P < 0.001. **D** Images and quantitative analysis of apoptotic OGD-stimulated SH-SY5Y cells incubating with culture medium and purified mitochondria from A1 astrocytes treated with Hep, EXO and EXO-Hep in TUNEL assay. The scale bar is 100 μm and applies to all Figure D, Data represent means ± SD (n = 3), **P < 0.01, ***P < 0.001. **E** Western blot analysis on the expressions of associated apoptotic proteins such as BAX and BCL-2 in OGD-stimulated SH-SY5Y cells treated with Mito/AS, Mito/A1-AS, Mito/A1-AS (EXO), Mito/A1-AS (Hep) and Mito/A1-AS (EXO-Hep). Data represent means ± SD (n = 3),*P < 0.05, **P < 0.01, ***P < 0.001

Since we previously demonstrated that mitochondrial function in neurons can be regulated by the transfer of astrocytic mitochondria into neurons, we further verified the potential role of exogenous astrocytic mitochondria in affecting survival fate of OGD-stimulated SH-SY5Y cells. To explore whether EXO-Hep improved the survival rate of neurons through mitochondrial transmission, apoptosis of OGD-stimulated SH-SY5Y cells was investigated after co-incubation with Mito/AS, Mito/A1-AS, Mito/A1-AS(Hep), Mito/A1-AS(EXO) and Mito/A1-AS (EXO-Hep) using MTT and TUNEL assays. As shown in Fig. 6B and D, compared to OGD-stimulated SH-SY5Y cells treated with CM/AS, culture medium containing mitochondria from A1-AS (CM/A1-AS) significantly reduced the viability of OGD-stimulated SH-SY5Y cells to 59.10% and enhanced the number of TUNEL-positive cells. In contrast, CM/A1-AS without the presence of mitochondria induced limited cytotoxicity of OGD-stimulated SH-SY5Y cells and obtained cell viability to 84.38% compared to cells treated with CM/A1-AS containing mitochondria. Furthermore, after treatment with culture medium containing mitochondria from A1-AS pretreated with Hep (CM/A1-AS(Hep)), EXO (CM/A1-AS(EXO)) and EXO-Hep (CM/A1-AS(EXO-Hep)), the viability of OGD-stimulated SH-SY5Y cells was enhanced. All results indicated that mitochondria secreted by astrocytes contributed to the variation of cell viability in OGD-stimulated SH-SY5Y cells. To further clarify the specific role of astrocytic mitochondria in regulating neurons, pure mitochondria were obtained from the culture medium of A1 astrocytes treated with Hep, EXO and EXO-Hep and further co-incubated with OGD-stimulated SH-SY5Y cells. It was found that Mito/A1-AS(Hep), Mito/A1-AS(EXO) and Mito/A1-AS (EXO-Hep) increased cell viability of OGD-stimulated SH-SY5Y cells and reduced the number of TUNEL-positive cells (Fig. 6C and D). In particular, Mito/A1-AS (EXO-Hep) resulted in the highest cell viability and lowest number of TUNEL-positive cells. Furthermore, we used a western blot assay to examine the expression of apoptotic proteins in OGD-stimulated SH-SY5Y cells. As shown in Fig. 5E, after treated with Mito/A1-AS, the expression of cytochrome c was decreased in mitochondria of OGD-stimulated SH-SY5Y cells. In addition, the higher expression of BAX and lower expression of BCL-2 were observed (Fig. 6E). It indicated that the cascade reaction of mitochondria mediated cellular apoptosis was activated. In contrast, after treated with Mito/A1-AS (EXO-Hep), the expression of mitochondrial cytochrome c in OGD-stimulated SH-SY5Y cells was enhanced (Fig. 5E), and mitochondrial-mediated neuronal apoptotic effects were inhibited by increasing the ratio of BCL-2/BAX, as shown in our western blot analyses (Fig. 6E). In conclusion, it suggested that with rupture of the mitochondrial membrane mediated by A1 astrocytic mitochondria, cytochrome c was rapidly released from mitochondria into the cytoplasm in neurons, which in turn aggravated cell apoptosis. EXO-Hep-mediated mitochondrial normalization in A1 astrocytes mitigated mitochondria-mediated damage to ODG-stimulated neurons and reduced the mortality of neurons.

### Neuroprotection of EXO-Hep in tMCAO rats

**Fig. 7:**
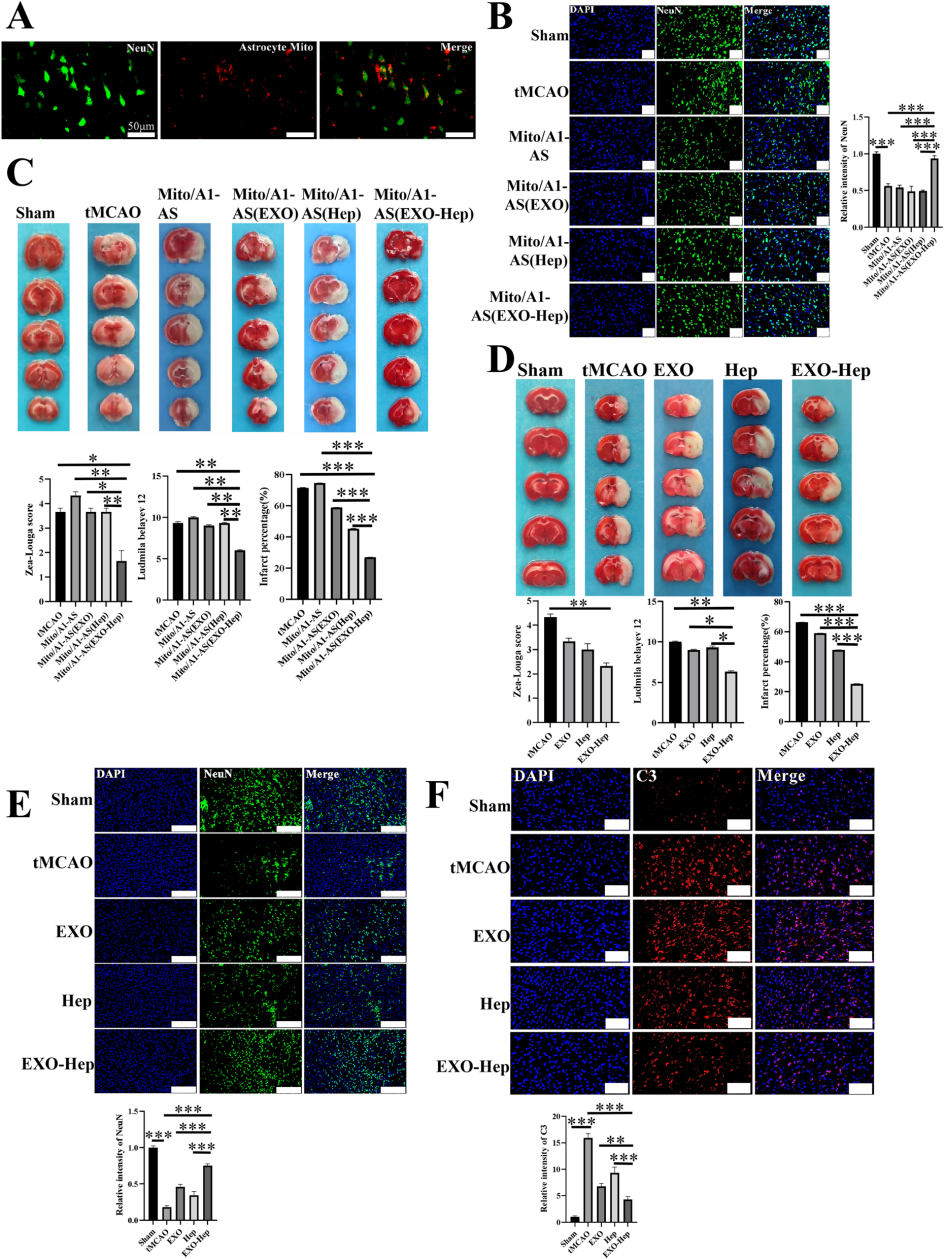
Neuroprotection of EXO-Hep in tMCAO rats. **A** After purified astrocytic mitochondria were labelled with Mito Tracker Red and injected into the cortex on the ischemic side of the rat, the colocalization of red labelled astrocytic mitochondria with neurons labelled with anti-NeuN (green) was observed by immunofluorescence staining. Scale scale is 50 μm. **B** Representative immunofluorescence staining and quantitative analysis for NeuN positive cells (green) after treated with purified mitochondria from A1-AS treated with Hep, EXO and EXO-Hep in ischemic cerebral tissue of tMCAO rats at 2 h of ischemia following by 24 h reperfusion. NeuN antibodies were used to stain neurons in ischemic cerebral tissue of tMCAO rats. DAPI (blue) was used as a nuclear marker. Quantification of the number of NeuN positive neurons were presented in panel. The relative intensity of NeuN was determined by calculating the ratio of fluorescent intensity of NeuN in groups to fluorescent intensity of NeuN in sham group. The scale bar in all Fig. 7B is 100 μm. Data are expressed as means ± SD (n = 3), ***P < 0.001. **C** Representative brain slices with infarcts were stained by 2,3,5-triphenyltetrazolium chloride (TTC) and infarct volume was calculated in tMCAO rats treated with purified mitochondria from A1-AS treated with Hep, EXO and EXO-Hep at 2 h of ischemia following by 24 h reperfusion. Data are expressed as means ± SD (n = 3), ***P < 0.001. In addition, Zea-Longa neurological scores and Ludmila Belayev neurological scores were determined. Data represent means ± SD (n = 3),*P < 0.05, **P < 0.01. **D** Representative brain slices with infarcts were stained by 2,3,5-triphenyltetrazolium chloride (TTC) and infarct volume was calculated in tMCAO rats treated with Hep, EXO and EXO-Hep at 2 h of ischemia following by 24 h reperfusion. Data are expressed as means ± SD (n = 3). ***P < 0.001. In addition, Zea-Longa neurological scores and Ludmila Belayev neurological scores were determined. Data represent means ± SD (n = 3), *P < 0.05, **P < 0.01. **E** Representative immunofluorescence staining and quantitative analysis for NeuN positive cells (green) in ischemic cerebral tissue of tMCAO rats treated with Hep, EXO and EXO-Hep at 2 h of ischemia following by 24 h reperfusion. NeuN antibodies were used to stain neurons in ischemic cerebral tissue of rats. DAPI (blue) was used as a nuclear marker. The relative intensity of NeuN was determined by calculating the ratio of fluorescent intensity of NeuN in groups to fluorescent intensity of NeuN in sham group. The scale bar in all Fig. 7E is 100 μm. Data are expressed as means ± SD (n = 3), ***P < 0.001. **F** Representative immunofluorescence staining and quantitative analysis for C3 positive cells (red) in ischemic cerebral tissue of tMCAO rats treated with Hep, EXO and EXO-Hep at 2 h of ischemia following by 24 h reperfusion. C3 antibodies were used to stain type A1 astrocytes in ischemic cerebral tissue of tMCAO rats. DAPI (blue) was used as a nuclear marker. The relative intensity of C3 was determined by calculating the ratio of fluorescent intensity of C3 in groups to fluorescent intensity of C3 in sham group. The scale bar in all Fig. 7F is 50 μm. Data are expressed as means ± SD (n = 3), **P < 0.01, ***P < 0.001

As we demonstrated that astrocytic mitochondria exerted a significant regulatory effect on the survival of neurons in vitro, we further clarified whether EXO-Hep mediated transfer of healthy astrocytic mitochondria into neurons protected against IS in a tMCAO model. First, astrocytic mitochondria were transplanted into the ischemic brain to evaluate their neuroprotection. Mitochondria in culture medium of astrocyte were collected and labelled with MitoTracker Red and directly injected into the peri-infarct cortex after 2 h of ischemia. Immunostaining results showed that MitoTracker Red-stained astrocytic mitochondria were located in neurons in ischemic region (Fig. 7A), suggesting an essential connection in astrocyte and neurons. As shown in Fig. 7B and C, after 24-h administration of mitochondria from A1 astrocytes, the percentage of infarct area and neurological scores were increased, and less NeuN-positive cells in ischemic penumbra were observed as compared to the untreated tMCAO group, indicating that implantation of damaged astrocytic mitochondria secreted by A1 astrocytes aggravated neuronal injury. In contrast, mitochondria from A1 astrocytes treated with EXO-Hep exerted a significant therapeutic effect on IS, as shown by the lower percentage of infarct area, decreased neurological scores and enhanced the number of NeuN-positive cells in ischemic penumbra compared to those in the tMCAO group treated with other formulations, revealing that EXO-Hep-induced healthy astrocytic mitochondria contributed to neuroprotection against IS. Similar to the results of astrocytic mitochondria-mediated neuroprotection against IS, following 2 h of occlusion, suture was subsequently removed to allow blood reperfusion. After reperfusion, 1 mL PBS, Hep, EXO and EXO-Hep containing Hep at a concentration of 0.75 mg/mL were administered via a single intravenous injection via the tail vein. It was found that the treatment with Hep, EXO and EXO-Hep significantly reduced the percent infarct area and decreased neurological scores. EXO-Hep exerted more powerful neuroprotective effects than that in free Hep and EXO treated tMCAO model by significantly decreasing the percent infarct area and reducing neurological scores (Fig. 7D). We next evaluated whether EXO-Hep treatment could decrease neuronal injury in ischemic penumbra. The number of neurons was observed and counted by immunofluorescence staining with the neurons marker NeuN. The qualitative analysis (Fig. 7E) revealed that more NeuN-positive cells were observed in ischemic penumbra in EXO-Hep treated tMCAO group than that in other groups. The result demonstrated that EXO-Hep treatment could effectively reduce neuronal injury after IS. Finally, we examined whether EXO-Hep reduced the activation of type A1 astrocytes by immunofluorescence staining (Fig. 7F). As expected, compared to tMCAO, EXO-Hep treatment reduced the activation of type A1 astrocytes. These results all indicated that EXO-Hep improved the mitochondrial function of astrocytes and contributed to the transfer of their healthy mitochondria to nearby damaged neurons, which in turn led to increased neuronal viability and neuroprotection against IS.

## Discussion

Mitochondria play a central role in the pathophysiological processes of acute IS. During the process of ischemic injury, mitochondria participate in the regulation of cell death by modulating ROS formation and calcium accumulation, controlling inflammation and inflammatory bodies [27, 28]. Excessive mitochondrial disruption during acute IS affects neurons by causing inefficient supply of ATP and releasing pro-apoptotic factors and excessive Ca^2+^ that kill neurons [29]. Mitochondria are the energy core of the electron transport chain that consumes oxygen and produces ATP, providing the energy necessary for life activities in cells and tissues [30]. It has long been believed that mitochondria remain in cells for life and are maternally inherited [31]. However, in some cases, mitochondria can be released outside of cells and transferred between cells [32]. A variety of cells have the ability to supply or receive mitochondria from other cells, including lymph, neurons or cardiomyocytes [33]. Studies have shown that the transfer of mitochondria from one cell to another is a modulatory mechanism responsible for regulating damaged cells from mitochondrial dysfunction in response to stress [34].

Our results demonstrated that the transfer of extracellular damaged or healthy mitochondria contributed to the detrimental or protective effects on IS. As shown by previous studies, in the central nervous system, there is extensive and complex information transmitted between astrocytes and neurons, which connects with neurons in a direct and interactive way and plays an important role in the development of the nervous system, synaptic transmission, regulating information processing and signal transmission, ion balance, and regulating meridian and synaptic plasticity. Astrocytes can directly provide functional mitochondria for neurons to rescue them from ischemic injury, indicating that the dynamic transfer process of mitochondria is not limited to inside cells but includes interactions between cells. Our new discovery revealed that activated type A1 astrocytes stimulated by LPS produced extracellular damaged mitochondria, and co-incubation with neurons caused neurons to undergo mitochondria-mediated apoptosis through the transfer of damaged fragmented mitochondria from astrocytes into neurons. Pathological mitochondrial disorder in astrocytes triggered neuronal damage by interrupting the mitochondrial function of neurons due to the transport of damaged mitochondria into neurons. After IS occurs, the structural and functional integrity of released astrocytic mitochondria is damaged by decreasing mitochondrial membrane potential, reducing ATP production and increasing ROS generation. Therefore, astrocytes can donate their harmful mitochondria to nearby damaged neurons, which in turn led to greater neuronal injury. It is reported that selective inhibition of Drp1/Fis1 interaction with Hep as a Drp1–Fis1 peptide inhibitor P110 reduced pathological mitochondrial fission, preserved cell survival and improved ATP production in a stroke model. Generally, lyophilized and hydrophilic Hep is stored at − 80 °C freezer and is dissolved in sterile water before use [35]. However, Hep as a hydrophilic protein drug still faces the limitation in poor physicochemical/biological stability, immunogenicity and crossing the physiological barrier [18]. In this study, we selected macrophage exosomes as multifunctional biomimetic drug delivery carriers to improve brain targeting ability of Hep. The results showed that compared to free Hep, EXO-Hep helped Hep cross the BBB and improved the targeted delivery of Hep in the ischemic brain and located Hep at activated astrocytes. EXO-Hep selectively inhibited mitochondrial pathological fission mediated by Drp1/Fis1 in astrocytes, improving the structural and functional integrity of released astrocytic mitochondria. As a result, more healthy astrocytic mitochondria were released and transported to adjacent neurons to support the survival of neurons.

Although we confirmed that EXO-Hep mediated neuronal protection via the exchange of mitochondria between astrocytes and neurons, we cannot ignore astrocyte-mediated inflammation as an important mechanism in the pathogenesis of IS. IS induced the transition of astrocytes from the resting state to the reactive state. Subsequently, cytokines, interleukins and other potentially cytotoxic molecules were released at higher levels from active astrocytes, thus destroying synaptic homeostasis and initiating neuronal injury [36]. Therefore, EXO-Hep-mediated inhibition of astrocyte activation and alleviation of astrocyte inflammation-mediated neuronal damage should be further explored in future studies.

Mitochondrial function in damaged cells can be regulated through endogenous or exogenous mitochondrial transfer, which has been confirmed in Parkinson's disease and spinal cord injury. Mitochondrial transfer provides a new treatment strategy conducive to the survival and regeneration of neurons after stroke. Before mitochondrial transfer is clinically utilized, a better understanding of the mechanisms of mitochondrial transmission is needed.

## Conclusions

Because fragmented and dysfunctional astrocytic mitochondria lead to neuronal damage during the process of IS, we prepared drug-loaded macrophage-derived exosomes to reduce mitochondrial dysfunction in type A1 astrocytes and promote the transfer of healthy astrocytic mitochondria into neurons to alleviate IS induced damage. These findings indicated that the application of EXO-Hep emphasized the efficacy of mitochondrial transfer therapy and may represent a promising alternative therapeutic approach for the treatment of IS.

## Data Availability

All data generated or analyzed during this study are included in this published article.

## Abbreviations

AS: Astrocytes
CNS: Central nervous system
Drp1: Dynamin-related protein 1
EXO- Hep: Heptapeptide-loaded macrophage-derived exosomes
Fis1: Fission 1
IS: Ischemic stroke
OGD: Oxygen–glucose deprivation
tMCAO: Transient middle cerebral artery occlusion
